# Mastic Kidney: A Rare Radiological Feature

**DOI:** 10.7759/cureus.77014

**Published:** 2025-01-06

**Authors:** Mariana Salvado de Morais, Sofia Cunha, Íris Galvão, Sara Lino, Fernando Maltez

**Affiliations:** 1 Internal Medicine, Centro Hospitalar Universitário de Lisboa Central, Lisbon, PRT; 2 Infectious Diseases, Centro Hospitalar Universitário de Lisboa Central, Lisbon, PRT

**Keywords:** extrapulmonary tuberculosis, flashcard, kidney imaging tuberculosis, mastic kidney, mycobacterium tuberculosis, renal tuberculosis, tuberculosis, urogenital tuberculosis

## Abstract

This case emphasizes the importance of considering tuberculosis (TB) in the differential diagnosis of complex kidney imaging findings. We report a case of a 43-year-old male patient from India who presented with a five-day history of fever and vomiting. Laboratory results revealed metabolic acidosis, anemia, acute kidney injury, and elevated inflammatory markers. A computed tomography urogram demonstrated significant findings: minimal parenchymal remnants in the left kidney replaced by caliectasis, ureteral pyelitis, and retroperitoneal lymphadenopathy, including calcified nodes in the right kidney. The diagnosis of genitourinary TB was made after bronchoalveolar lavage and urine samples confirmed the presence of *Mycobacterium tuberculosis*. The imaging findings were consistent with "mastic kidney," a rare radiological sign of renal TB, characterized by extensive parenchymal destruction. First-line antituberculous therapy was initiated, leading to clinical improvement. This case highlights the critical role of early diagnosis and the importance of considering TB in patients with multisystem involvement. "Mastic kidney," though rare, remains a key radiological sign of renal TB, underscoring the need for heightened clinical awareness in suspected cases of extrapulmonary TB.

## Introduction

Tuberculosis (TB) remains a significant global health challenge, with extrapulmonary manifestations accounting for up to 20% of all TB cases [1]. Among these, urogenital TB is the third most common form of extrapulmonary TB, presenting diagnostic challenges due to its often nonspecific symptoms [2]. Advances in imaging have become crucial for identifying such cases, particularly in endemic regions where delayed diagnosis can lead to extensive organ damage.

This case report highlights a rare radiological finding, i.e., "mastic kidney," a hallmark of renal TB, emphasizing the importance of clinical vigilance and the role of imaging in early detection and management [3].

## Case presentation

A 43-year-old male patient, originally from India and residing in Portugal for the past six months, presented to the emergency department with a five-day history of fever (vespertine, with a maximum temperature of 40ºC) and vomiting. The patient had no significant medical history, including a negative test for human immunodeficiency virus (HIV), and denied other symptoms such as weight loss, night sweats, cough, dyspnea, dysuria, or lower back pain. On clinical examination, positive findings included a thin, pale, polypneic, and diaphoretic appearance, along with enlarged lymph nodes in the cervical, axillary, and inguinal regions. Laboratory tests revealed metabolic acidosis, microcytic hypochromic anemia, acute kidney injury, hyponatremia, and elevated inflammatory markers (Table 1).

**Table 1:** Laboratory tests.

Parameter	Observed value	Reference range
pH	7.195	7.35–7.45
Partial pressure of carbon dioxide (pCO₂)	22.7	35–45 mmHg
Partial pressure of oxygen (pO₂)	132	75–100 mmHg
Bicarbonate (HCO₃)	8.7	22–28 mEq/L
Lactate	0.5	0.5–2.0 mmol/L
Creatinine	7.51	0.6–1.2 mg/dL
Sodium (Na⁺)	130	135–145 mEq/L
Leukocytes	9.96	4.5–11 (10⁹/L)
C-reactive protein	148.9	<5.0 mg/L
Procalcitonin	1.45	<0.05 ng/mL

**Figure 1:**
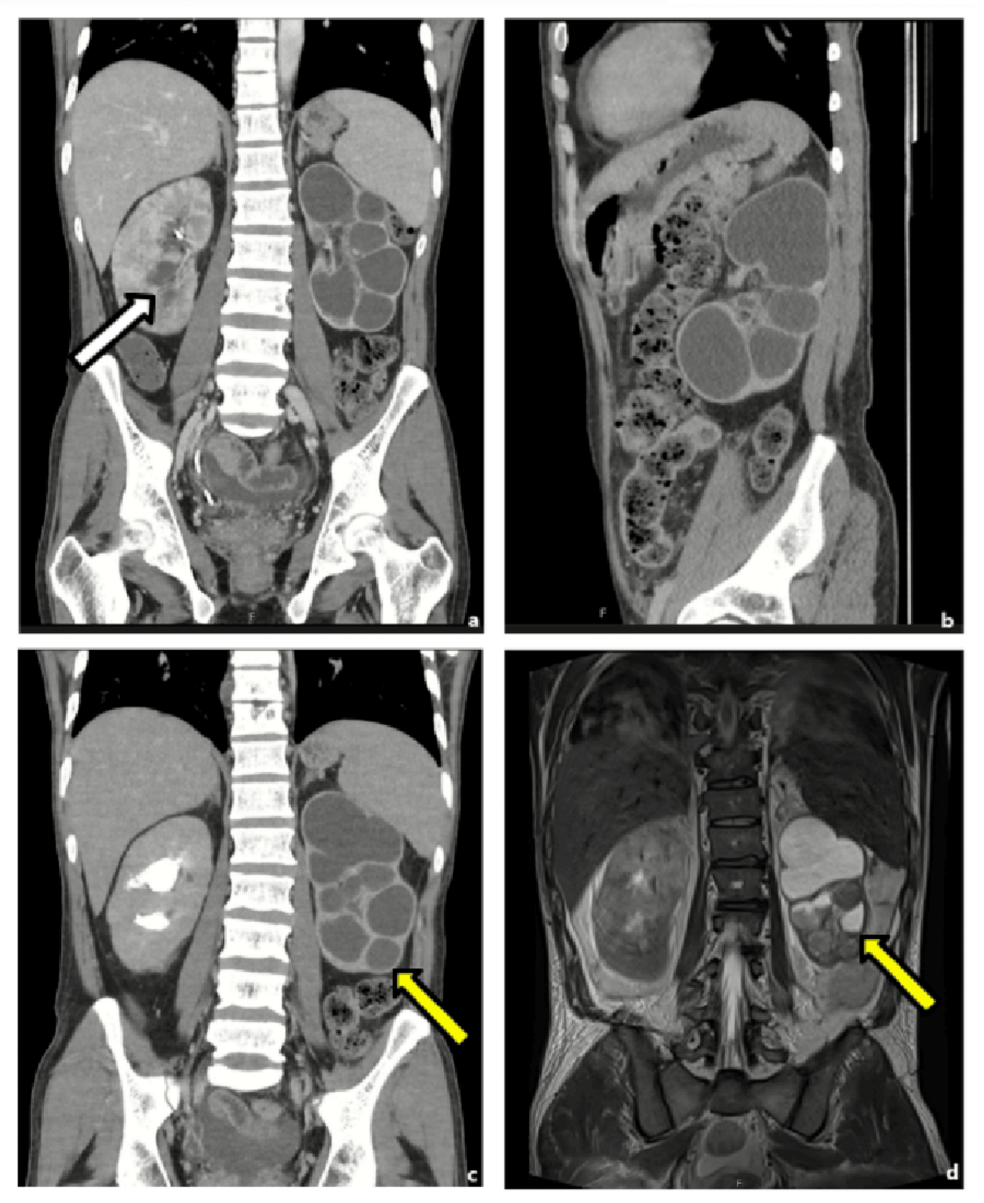
CT urogram in the coronal (a) and sagittal (b) plane after contrast admin in the portal phase and in the coronal plane in the excretory phase (c), and T20weighted MRI on the coronal plane (d). The left kidney exhibited minimal residual parenchyma extensively replaced by apparent caliectasis (yellow arrow), while the right kidney displayed ureteral pyelitis (white arrow).

A computed tomography (CT) urography (Figure 1) demonstrated significant findings: the left kidney exhibited minimal residual parenchyma extensively replaced by apparent caliectasis (yellow arrow), while the right kidney displayed ureteral pyelitis (white arrow) with marked retroperitoneal lymphadenopathy, including calcified lymph nodes.

The patient underwent right ureteral stent placement due to chronic pyelonephritis with obstructive uropathy. Systemic findings included lung parenchymal changes characterized by a miliary pattern, as well as soft tissue and bone lesions at the D9 and D10 vertebral levels.

Given the endemic prevalence in India and the systemic involvement, genitourinary TB was considered. *Mycobacterium tuberculosis *complex was isolated from both bronchoalveolar lavage and urine samples. Bronchoalveolar lavage revealed acid-fast bacilli (negative in expectorated sputum). Magnetic resonance imaging was performed to assess spinal involvement, revealing findings suggestive of tuberculous spondylodiscitis.

First-line antituberculous therapy (isoniazid, rifampin, pyrazinamide, ethambutol) was initiated, leading to clinical improvement. Susceptibility testing showed sensitivity to these medications. The patient was discharged after 44 days and continued follow-up with multiple consultations in nephrology, urology, and infectious diseases and at the Pulmonological Diagnostic Center for Tuberculosis. Despite improvements in renal function, the patient developed multiple strictures due to urinary TB, necessitating continuous catheterization.

Post-discharge, he experienced recurrent pyelonephritis requiring two hospital readmissions, one complicated by* Escherichia coli *bacteremia. Repeat magnetic resonance imaging demonstrated extensive urinary system changes, including prostate, seminal vesicle, bladder, and kidney involvement. The findings were attributed to prior TB, with marked atrophy of the left kidney due to chronic obstructive nephropathy and scarring changes in the right kidney. The patient has now completed one year of treatment with antituberculous medications. Currently, the kidney has shown clear improvement (urea = 68 mg/dL, creatinine = 2.64 mg/dL), and the ureteral stent was retained.

## Discussion

Urogenital TB is the third most common form of extrapulmonary TB, presenting diagnostic challenges due to its nonspecific symptoms and varied radiological features [2]. Renal TB is particularly concerning in endemic areas, where delayed diagnosis may lead to significant morbidity and irreversible organ damage.

This case illustrates a rare radiological finding known as "mastic kidney," typically associated with advanced renal TB. The term is derived from the Greek word "mastiche," referring to mastic tree resin, as the radiological appearance resembles a shrunken, irregularly shaped kidney with extensive parenchymal destruction and caliectasis. This feature, though rare, serves as a critical clue in diagnosing renal TB [4,5].

In this patient, imaging findings prompted a differential diagnosis of genitourinary TB. The presence of calcified lymph nodes supported the suspicion, as calcifications often indicate chronic infections such as TB. Systemic involvement, including lung changes, vertebral osteomyelitis, and soft tissue damage, corroborated the diagnosis. The positive identification of *Mycobacterium tuberculosis* in bronchoalveolar lavage and urine samples confirmed the diagnosis and provided a basis for definitive treatment.

This case underscores the necessity of high clinical suspicion in regions with a high TB burden, especially when patients present with systemic symptoms and nonspecific findings. Advanced imaging techniques such as CT and MRI are invaluable for diagnosing complex TB cases [3]. These modalities not only aid in diagnosis but also assess organ involvement and guide treatment planning. A multidisciplinary approach integrating clinical, radiological, and microbiological data ensures accurate and timely diagnosis.

Early intervention with first-line antituberculous therapy proved effective in this case, emphasizing the need for prompt diagnosis and treatment to prevent irreversible complications such as end-stage renal failure.

## Conclusions

The presentation of "mastic kidney," a rare radiological hallmark of advanced renal TB, underscores the critical role of imaging in diagnosing atypical TB cases. The patient's systemic involvement illustrates TB's potential to mimic other conditions, emphasizing the need for heightened clinical suspicion in endemic regions.

This case serves as a reminder that TB should remain a differential diagnosis in patients from endemic regions with unexplained systemic symptoms. Recognizing TB's myriad presentations and employing advanced diagnostics can ensure prompt identification and management, improving patient outcomes and reducing long-term complications. Early and accurate diagnosis, coupled with timely initiation of first-line antituberculous therapy, remains essential in preventing irreversible organ damage and improving patient outcomes. This report highlights TB's enduring relevance in global health and the indispensable role of modern diagnostics in managing this ancient disease.
